# Abnormality-aware multimodal learning for WSI classification

**DOI:** 10.3389/fmed.2025.1546452

**Published:** 2025-02-25

**Authors:** Thao M. Dang, Qifeng Zhou, Yuzhi Guo, Hehuan Ma, Saiyang Na, Thao Bich Dang, Jean Gao, Junzhou Huang

**Affiliations:** ^1^Department of Computer Science and Engineering, University of Texas at Arlington, Arlington, TX, United States; ^2^Department of Pulmonary and Critical Care, University of Arizona, Phoenix, AZ, United States

**Keywords:** WSI analysis, multimodal fusion, abnormal detection, foundation model, Gaussian Mixture Variational Autoencoder

## Abstract

Whole slide images (WSIs) play a vital role in cancer diagnosis and prognosis. However, their gigapixel resolution, lack of pixel-level annotations, and reliance on unimodal visual data present challenges for accurate and efficient computational analysis. Existing methods typically divide WSIs into thousands of patches, which increases computational demands and makes it challenging to effectively focus on diagnostically relevant regions. Furthermore, these methods frequently rely on feature extractors pretrained on natural images, which are not optimized for pathology tasks, and overlook multimodal data sources such as cellular and textual information that can provide critical insights. To address these limitations, we propose the **A**bnormality-**A**ware **M**ulti**M**odal (AAMM) learning framework, which integrates abnormality detection and multimodal feature learning for WSI classification. AAMM incorporates a Gaussian Mixture Variational Autoencoder (GMVAE) to identify and select the most informative patches, reducing computational complexity while retaining critical diagnostic information. It further integrates multimodal features from pathology-specific foundation models, combining patch-level, cell-level, and text-level representations through cross-attention mechanisms. This approach enhances the ability to comprehensively analyze WSIs for cancer diagnosis and subtyping. Extensive experiments on normal-tumor classification and cancer subtyping demonstrate that AAMM achieves superior performance compared to state-of-the-art methods. By combining abnormal detection with multimodal feature integration, our framework offers an efficient and scalable solution for advancing computational pathology.

## 1 Introduction

Accurate cancer diagnosis and quantitative evaluation heavily depend on pathologists examining tissue samples through pathological images (1, 2). Recent advances in digital slide scanning, deep learning, and the availability of large datasets have revolutionized computational pathology. These developments enable the use of whole slide images (WSIs) from Hematoxylin and Eosin (H&E)-stained specimens (3) for tasks such as cancer classification (4–6), cell detection (7), and cell segmentation (8–10). However, the gigapixel resolution of WSIs, combined with the lack of pixel-level annotations, poses significant challenges. Developing efficient and effective methods for analyzing such high-resolution WSIs is crucial to advancing cancer diagnosis and prognosis (11).

Analyzing gigapixel WSIs poses significant challenges due to their massive size and lack of pixel-level annotations. Most existing methods rely on a standard pipeline, which involves dividing WSIs into numerous patches, extracting patch-level features using models pretrained on ImageNet, and training a slide-level classifier to aggregate these features for prediction. While effective in some cases, this pipeline struggles with several limitations. WSIs with only slide-level labels must be divided into thousands of small patches due to their massive size. Moreover, pretrained feature extractors are designed for natural images, which means their embeddings are not well-suited for the unique characteristics of pathology data. Additionally, existing approaches predominantly focus on visual data, missing opportunities to leverage other complementary modalities, such as cellular information or textual descriptions, which can provide deeper insights for cancer diagnosis and classification. While recent pathology-specific foundation models have made significant advancements in providing high-quality representation features for WSI analysis, challenges persist in efficiently selecting diagnostically relevant regions and effectively fusing multimodal information.

To overcome these challenges, we propose a novel framework that integrates abnormality detection with multimodal feature learning. To address the issue of processing numerous patches, we introduce an abnormal detection (AD) module based on a Gaussian Mixture Variational Autoencoder (GMVAE) (12). The AD module, trained exclusively on normal WSIs, identifies abnormal patches by detecting deviations from the normal tissue distribution. This enables the framework to focus on the most informative patches, reducing computational overhead and mitigating issues caused by an imbalanced distribution of positive and negative regions (13). Our framework also tackles the limitations of unimodal feature extraction by leveraging pathology-specific foundation models. These models generate features aligned with pathology tasks and support multimodal data integration. For instance, cell-level segmentation techniques (14) provide localized cellular insights, while large language models (LLMs) generate textual descriptions of pathology images (15), offering additional context. However, simple concatenation of features from different modalities often results in sparse and noisy representations, limiting their utility. To address this, we introduce a three-step cross-attention module that effectively integrates patch-level, cell-level, and text-level features, enabling a comprehensive representation for cancer diagnosis and classification.

We present the **A**bnormality-**A**ware **M**ulti**M**odal (AAMM) learning framework for WSI classification, which integrates these components into a unified pipeline. The framework first employs the GMVAE-based abnormal detection module to select the top-*k* patches from gigapixel WSIs. Multimodal embeddings are then generated from these selected patches using foundation models for image, cell, and text-based features. Finally, these multimodal features are fused using the cross-attention module to enable robust classification. Our contributions can be summarized as follows:

- We introduce a GMVAE-based abnormal detection module that naturally and efficiently selects top-*k* informative patches, reducing computational costs and enhancing the learning of patch-level features for abnormal detection.
- We propose a novel multimodal framework that integrates image, cell, and text-based features extracted from pathology foundation models, achieving superior performance on both cancer classification and subtyping datasets.
- We conduct comprehensive experiments on multiple datasets for normal-tumor classification and cancer subtyping, demonstrating that AAMM significantly outperforms state-of-the-art (SOTA) methods.

This work builds upon our previous conference paper (16), which is presented at *the 15th ACM Conference on Bioinformatics, Computational Biology, and Health Informatics* (ACM BCB 2024). In this extended version, we provide a more comprehensive background introduction, an expanded method description, and an in-depth discussion of experimental results. Additionally, we significantly improve and extend the previous work in three main aspects: 1) We introduce an improved abnormal detection module based on a Gaussian Mixture Variational Autoencoder (GMVAE), which offers a more robust capability to capture the inherent variability of normal tissue and detect deviations indicative of abnormalities. 2) We implement a weighted cross-entropy loss for the classifier to address class imbalance and achieve better performance, particularly for underrepresented classes. 3) We present extensive new experimental results, including detailed ablation studies and improved visualizations, to provide deeper insights into the effectiveness of the proposed AAMM framework.

## 2 Related work

### 2.1 Multiple instance learning for WSI analysis

We adopt the MIL approach for WSI classification, as MIL effectively handles large data with only slide-level labels, given that obtaining instance-level annotations in medical imaging is costly and time-consuming. Particularly, each WSI is treated as a bag of multiple instances. A bag is labeled as *Y*_*i*_ = 1 if $\overset{m}{\underset{j=1}{\sum }}y_{i,j}\geq 1$, meaning it contains at least one positive instance such as a tumor-cropped patch, and *negative* when $\overset{m}{\underset{j=1}{\sum }}y_{i,j}=0$.

Current MIL methods can be broadly categorized into two types: bag-level and instance-level. Instance-level MIL emphasizes learning directly at the instance level, and derives bag-level predictions by simply aggregating these instance predictions with Mean or Max-pooling (17, 18). Bag-level MIL, on the other hand, transforms instances into low-dimensional embeddings, aggregating these into bag-level representations for analytical tasks. The bag-level MIL performs better because this kind of modeling involves less inductive bias than the instance-level MIL processing with set weights. ABMIL (19) uses attention weights to learns the weights of instance representations adaptively, significantly improving robustness. However, it treats patches independently, which limits its ability to capture contextual interactions. CLAM (20) further enhances ABMIL by incorporating a clustering constraint, which pulls the most and least attended instances apart. Despite these improvements, CLAM and similar MIL-based approaches typically treat different patches independently and do not account for potential cross-interactions, limiting their ability to become context-aware. DSMIL (21) integrates self-supervised contrastive learning and non-local operations to model relations, improving accuracy but potentially introducing noisy signals and high computational demands. TransMIL (22) utilizes transformer-based MIL to model interactions between instances. Its key component, the Pyramid Position Encoding Generator (PPEG), requires adding additional embeddings to ensure the number of instances in a bag is a square number, leading to redundancy and potentially incorrect weighting of patch importance. Dual-Query MIL (23) combines MIL and self-attention with dynamic meta-embedding, decoupling input size from latent representation (24) but introducing computational overhead due to fine-tuning needs. We follow bag-level MIL as the implementation in this study.

### 2.2 Pathology foundation model

Following the advent of foundation models in computer vision (25–27) and natural language processing (28, 29), new research has looked at the creation of foundation models in histology based on self-supervised learning (30, 31), image-text learning (32), segment model (14, 25), and multimodal large language model (33). GigaPath (31), pre-trained on 1.3 billion image patches, excels in cancer subtyping and mutation prediction by integrating local and global features. UNI (30) uses self-supervised learning to extract features from unlabeled data, significantly improving prediction accuracy and reducing reliance on labeled data. CONCH (32) enhances breast cancer diagnosis by combining pathology images with clinical data to generate detailed textual descriptions and reports. Trained on over 1.17 million image-text pairs, CONCH performs tasks like image captioning and text-to-image retrieval, making it versatile in clinical settings. The Segment Anything Model (SAM) (25) is a versatile vision segmentation model that creates detailed segmentation masks from user prompts. Trained on over 1 billion masks from 11 million images, SAM excels in zero-shot performance on new tasks. MedSAM (34), an adaptation for medical image segmentation, is trained on over 1.5 million medical image mask pairs and excels in segmenting diverse anatomical structures and lesions, outperforming specialist models. Segment Any Cell (SAC) (14) fine-tunes SAM for nuclei segmentation in biomedical images, using Low-Rank Adaptation (35) in the attention layer to handle complex nuclei segmentation tasks effectively. LLaVA-Med (33), adapted for biomedical applications through a two-stage training process with extensive visual and textual data, significantly improves tasks like medical visual question answering (VQA) by leveraging domain-specific knowledge for better performance in both open and closed-set questions. The Quilt-LLaVA model (15), using the Quilt-1M dataset (36) with 1 million paired image-text samples and the Quilt-Instruct dataset for instruction tuning, excels in reasoning about histopathology images, providing detailed spatial localization and context-based analysis for enhanced performance in identifying abnormalities and describing images. In this study, we employ CONCH (32), Segment Any Cell (SAC) (14), and Quilt-LLaVA (15) as the implemented foundation models for each level features.

## 3 Methodology

**Figure 1:**
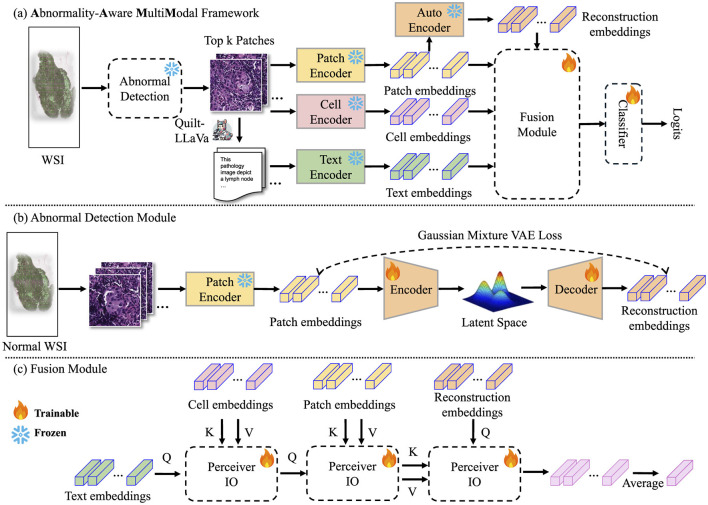
Pipeline of our proposed method. **(A)** AAMM framework: This framework processes input images by extracting patch-level features through an encoder. These features are analyzed using a pre-trained Abnormal Detection module, which selects the top-*k* potential patches by measuring reconstruction errors, flagging instances with significant abnormalities for further analysis. The selected instances are then used to extract cell-level and text-level features, which are fused with patch and reconstruction features via cross-attention mechanisms before being classified to predict the final label. **(B)** Abnormal Detection module: The process begins by dividing normal WSIs into patches and extracting patch-level embeddings using an image encoder. These embeddings are utilized to train the Abnormal Detection module. **(C)** Fusion module: The selected patch-cell-text-reconstruction embeddings are integrated using cross-attention mechanisms to combine information from multiple modalities. The resulting fused embeddings are averaged into a bag-level representation.

In this section, we first provide the formulation of our proposed AAMM. Then, we briefly introduce MIL and autoencoder-based abnormal detection, and explain why these concepts are well-suited for WSI-based tumor classification tasks. Finally, we describe AAMM in detail. Algorithm 1 summarizes the detailed implementation, and Figure 1 illustrates the workflow of our proposed AAMM.

**Algorithm 1:**
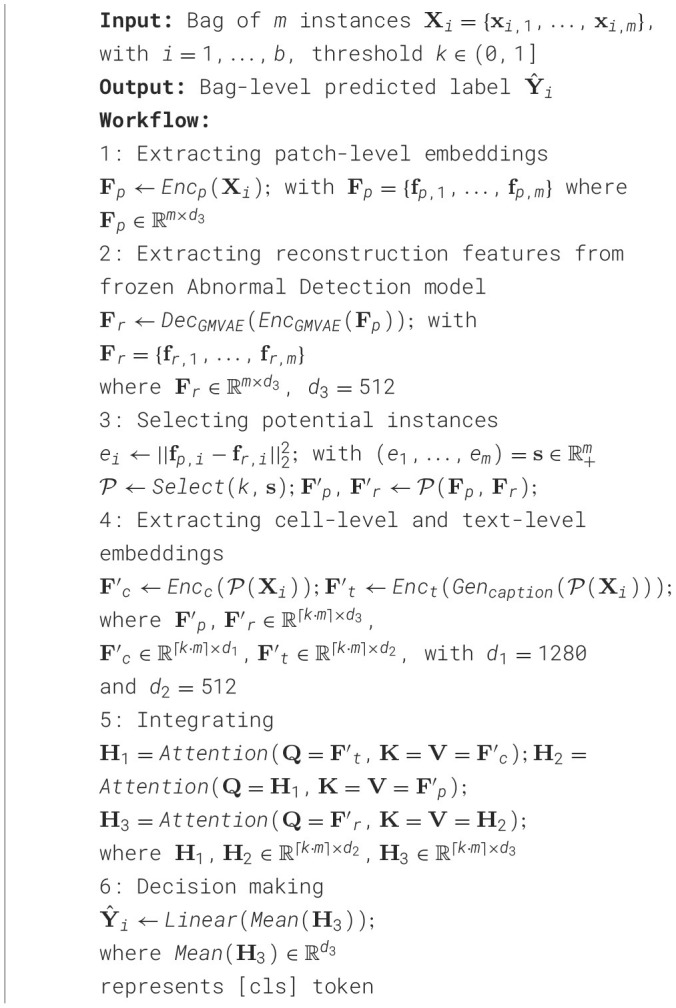
Abnormality-Aware Multimodal Learning Framework.

### 3.1 Problem formulation

We consider a dataset $D={\left\{\left({\text{X}}_{i},{\text{Y}}_{i}\right)\right\}}_{i=1}^{b}$ consisting of *b* bags, where each bag **X**_*i*_ contains a variable number of instances *m*_*i*_, formally defined as **X**_*i*_ = {**x**_*i*,1_, **x**_*i*,2_, …, **x**_*i*,_*m*_*i*___} for *i* = 1, …, *b*. Each bag **X**_*i*_ is associated with a binary label **Y**_*i*_ ∈ {0, 1}, while the instance-level labels ${\left\{y_{i,j}\right\}}_{j=1}^{m_{i}}$ for each **x**_*i,j*_ are unknown. The primary objective is to develop a prediction function $f\left({\text{X}}_{i}\right)={\hat{\text{Y}}}_{i}$ that accurately estimates the true bag-level label **Y**_*i*_ for all bags, such that ${\hat{\text{Y}}}_{i}={\text{Y}}_{i}$ for *i* = 1, …, *b*, without access to the individual instance-level labels ${\left\{y_{i,j}\right\}}_{j=1}^{m_{i}}$.

In the context of pathology images, each bag **X**_*i*_ represents a WSI, and each instance **x**_*i,j*_ corresponds to a cropped image patch extracted from the WSI. Only the bag-level labels **Y**_*i*_ are available, indicating the presence or absence of a particular condition (e.g., malignancy) within the entire WSI, whereas the labels for individual patches ${\left\{y_{i,j}\right\}}_{j=1}^{m_{i}}$ are not provided. Typically, the number of positive patches that contain the condition of interest is significantly smaller than the number of negative patches within each WSI. To address this imbalance and reduce computational redundancy, we define a selection function $Select\left(\cdot \right):{\text{X}}_{i}\to P_{i}$, where $P_{i}\subseteq {\text{X}}_{i}$ denotes a subset of potential patches. This function is designed to thoughtfully select a representative subset of patches that are more likely to contain the condition of interest, thereby guiding the MIL process to focus on the most informative instances. Formally, the selection function can be expressed as $P_{i}=Select\left({\text{X}}_{i}\right)=\left\{{\text{x}}_{i,j}|j\in J_{i}\right\}$, where $J_{i}\subseteq \left\{1,\ldots ,m_{i}\right\}$ is an index set determined by criteria such as reconstruction error thresholds. By applying this selection mechanism, the MIL framework (i.e., Section 2.1) operates on a reduced and more relevant set of instances $P_{i}$, thereby enhancing both the efficiency and effectiveness of the bag-level label prediction. This approach mitigates the challenges posed by the predominance of negative patches and facilitates more accurate and computationally feasible learning within the MIL paradigm.

### 3.2 Abnormal detection module

Detecting abnormalities in histopathological images involves identifying patterns that differ from those found in normal instances. Variational Autoencoders (VAEs) (37) are well-suited to this anomaly detection task. By training on normal data only, a VAE learns a distribution *p*_θ_(**z**) over the latent space that captures the variability of normal features. When given abnormal data, the reconstruction error increases, indicating it differs from the learned normal distribution.

#### 3.2.1 Variational autoencoder

A vanilla VAE consists of an encoder *Enc*_*VAE*_ and a decoder *Dec*_*VAE*_. Given an input **x** ∈ ℝ^*D*^ (e.g., a cropped patch from WSI), the encoder produces a latent distribution:


(1) $$\begin{array}{l} q_{\phi }\left(\text{z}|\text{x}\right)=N\left(\text{z};\mu \left(\text{x}\right),\sigma^{2}\left(\text{x}\right)\text{I}\right). \end{array}$$

To enable gradient-based optimization, we use the reparameterization strategy:


(2) $$\begin{array}{l} \text{z}=\mu \left(\text{x}\right)+\sigma \left(\text{x}\right)⊙\epsilon ,\;\epsilon \sim N\left(0,\text{I}\right), \end{array}$$

and then reconstruct the input as $\hat{\text{x}}\leftarrow Dec_{VAE}\left(\text{z}\right)$, such that $\hat{\text{x}}\approx \text{x}$. The VAE is trained by minimizing the negative Evidence Lower BOund (ELBO):


(3) $$\begin{array}{l} L_{VAE}\left(\text{x}\right)=-{𝔼}_{q_{\phi }\left(\text{z}|\text{x}\right)}\left[\log p_{\theta }\left(\text{x}|\text{z}\right)\right]+KL\left(q_{\phi }\left(\text{z}|\text{x}\right)\|p_{\theta }\left(\text{z}\right)\right), \end{array}$$

with a prior $p_{\theta }\left(\text{z}\right)=N\left(0,\text{I}\right)$ and likelihood $p_{\theta }\left(\text{x}|\text{z}\right)=N\left(\text{x};\hat{\text{x}},\text{I}\right)$. Under these Gaussian assumptions, the reconstruction term can be approximated by a mean squared error (MSE), and the Kullback-Leibler divergence (KLD) term can be expressed as follows:


(4) $$\begin{array}{l} L_{MSE}=\frac{1}{N}\overset{N}{\underset{i=1}{\sum }}\|{\text{x}}_{i}-\hat{{\text{x}}_{i}}{\|}_{2}^{2}, \end{array}$$

(5) $$\begin{array}{l} L_{KLD}=\frac{1}{N}\overset{N}{\underset{i=1}{\sum }}\left[-\frac{1}{2}\overset{D}{\underset{j=1}{\sum }}\left(1+\log\left(\sigma_{i,j}^{2}\right)-\mu_{i,j}^{2}-\sigma_{i,j}^{2}\right)\right]. \end{array}$$

By training the VAE solely on *N* patches **x** = **f**_*p*_ from a dataset $B={\left({\text{X}}_{i},{\text{Y}}_{i}\right)}^{b}$ using the objective function $L_{VAE}=L_{MSE}+L_{KLD}$, the latent space is shaped to model only normal variations. Consequently, abnormal instances that diverge from this learned distribution yield higher reconstruction errors, thereby enabling the detection of abnormalities.

#### 3.2.2 Gaussian mixture VAE

A single Gaussian prior may be insufficient to represent the complex distributions arising from diverse tissue morphologies in WSI data. To address this, we applied the Gaussian Mixture VAE (GMVAE) (12), which replaces the single Gaussian prior with a mixture of *K* Gaussian:


(6) $$\begin{array}{l} p_{\theta }\left(\text{z}\right)=\overset{K}{\underset{k=1}{\sum }}\pi_{k}N\left(\text{z}|\mu_{k},\sigma_{k}^{2}\text{I}\right), \end{array}$$

where {π_*k*_}, {**μ**_*k*_}, and $\left\{\sigma_{k}^{2}\right\}$ define the mixture weights, means, and variances, respectively. The GMVAE objective still follows the ELBO's form. Choosing, for simplicity, $q_{\phi }\left(\text{z}|\text{x}\right)=N\left(\text{z};\mu \left(\text{x}\right),\sigma^{2}\left(\text{x}\right)\text{I}\right)$, the GMVAE loss takes a form similar to the VAE, but now the prior is a mixture:


(7) $$\begin{array}{l} L_{GMVAE}\left(\text{x}\right)=-{𝔼}_{q_{\phi }\left(\text{z}|\text{x}\right)}\left[\log p_{\theta }\left(\text{x}|\text{z}\right)\right]+KL\left(q_{\phi }\left(\text{z}|\text{x}\right)\|p_{\theta }\left(\text{z}\right)\right). \end{array}$$

As before, the first term reduces to the reconstruction cost ($L_{MSE}$). The second term expands as:


(8) $$\begin{array}{l} KL\left(q_{\phi }\left(\text{z}|\text{x}\right)\|p_{\theta }\left(\text{z}\right)\right)={𝔼}_{q_{\phi }\left(\text{z}|\text{x}\right)}\left[\log q_{\phi }\left(\text{z}|\text{x}\right)-\log p_{\theta }\left(\text{z}\right)\right]. \end{array}$$

Since we have:


(9) $$\begin{array}{l} \log q_{\phi }\left(\text{z}|\text{x}\right)=-\frac{D}{2}\log\left(2\pi \right)-\frac{1}{2}\overset{D}{\underset{j=1}{\sum }}\left(\frac{{\left(z_{j}-\mu_{j}\right)}^{2}}{\sigma_{j}^{2}}+\log\left(\sigma_{j}^{2}\right)\right), \end{array}$$

and


(10) $$\begin{array}{l} \log p_{\theta }\left(\text{z}\right)=\log\left(\overset{K}{\underset{k=1}{\sum }}\pi_{k}N\left(\text{z};\mu_{k},\sigma_{k}^{2}\text{I}\right)\right), \end{array}$$

the objective encourages *q*_ϕ_(**z**|**x**) to align with one of the mixture components, allowing the latent space to cluster and represent multiple modes of normal variation. Thus, we have:


(11) $$\begin{array}{l} L_{GMVAE}=L_{MSE}+{𝔼}_{q_{\phi }\left(\text{z}|\text{x}\right)}\left[\log q_{\phi }\left(\text{z}|\text{x}\right)-\log p_{\theta }\left(\text{z}\right)\right]. \end{array}$$

Once the GMVAE is trained and has learned a latent representation of normal patches, we freeze its parameters. Given a patch **f**_*p*_, we obtain its reconstruction embedding:


(12) $$\begin{array}{l} {\text{f}}_{r}=Dec_{GMVAE}\left(Enc_{GMVAE}\left({\text{f}}_{p}\right)\right). \end{array}$$

These embeddings are then integrated into the AAMM model. By leveraging the GMVAE's richer latent structure, subsequent classification and analysis tasks achieve improved performance in detecting and characterizing abnormalities in histopathological images.

### 3.3 Multimodal feature extraction

In this study, we consider each bag $\text{X}={\left\{{\text{x}}_{i}\right\}}_{i=1}^{m}$ as a collection of image patches, each measuring 1024 × 1024 pixels, from which we derive a rich set of multimodal features. We begin by processing each patch **x**_*i*_ using the CONCH (32) foundation model, which employs a ViT-Base-16 backbone, to obtain a patch-level embedding ${\text{f}}_{p}\in {\mathbb{R}}^{512}$. Letting **F**_*p*_ = {**f**_*p*,1_, …, **f**_*p,m*_} denote the set of all patch-level embeddings, we then apply a GMVAE-based abnormality detection module to produce reconstructed embeddings **F**_*r*_ = {**f**_*r*, 1_, …, **f**_*r,m*_}, with ${\text{f}}_{r}\in {\mathbb{R}}^{512}$. Evaluating the reconstruction error $e_{i}=\|{\text{f}}_{p,i}-{\text{f}}_{r,i}{\|}_{2}^{2}$ enables the identification of patches exceeding a predefined threshold *k*, thus selecting them for further cell and text feature extraction steps.

For each selected patch, we further enrich our representations by extracting cell-level features ${\text{f}}_{c}\in {\mathbb{R}}^{1280}$. To achieve this, we leverage a segmentation-adapted ViT encoder from the SAC model (14). In addition, we integrate textual context to enhance interpretability. Using the Quilt-LLaVA (15) foundation model, we generate descriptive phrases for each patch. These text descriptions of arbitrary length are then encoded by the CONCH model's text encoder to obtain fixed-size text-based embeddings ${\text{f}}_{t}\in {\mathbb{R}}^{512}$.

The prompts for the CAMELYON16, TCGA-Lung, and SLN-Breast datasets emphasize histopathological attributes, including visible features, tumor presence, and diagnostic properties. To generate patch descriptions with Quilt-LLaVA, we utilize prompts designed for short conversations, detailed descriptions, and complex medical reasoning. These prompts are adapted with relevant medical terms based on the tumor or subtype classification tasks, with response lengths limited to a maximum of 1024 tokens.

The prompts used for each dataset are as follows:

“*Can you describe the main features visible in this histopathology image? In a few words, what does the histopathology image depict? Is there a tumor in this pathology image? Are there abnormal, neoplastic, atypical, or metastatic cells in this pathology image? Make a diagnosis based on this single patch of histopathology image*.”

“*Can you describe the main features visible in this histopathology image? In a few words, what does the histopathology image depict? Is it lung adenocarcinoma or lung squamous cell carcinoma?*”

“*Can you describe the main features visible in this histopathology image? In a few words, what does the histopathology image depict? Is it positive or negative for breast carcinoma?*”

Finally, each selected patch is represented by the multimodal set {**f**_*p*_, **f**_*r*_, **f**_*c*_, **f**_*t*_}, collectively capturing visual, reconstructive, cellular, and textual cues. These comprehensive embeddings facilitate robust bag-level classification by leveraging multiple information streams to enhance decision-making.

### 3.4 Abnormality-aware multimodal learning

**Feature encoding with foundation models** Given *m* cropped patches from the bag **X**_*i*_, we derive cell features ${\text{F}}_{c}\in {\mathbb{R}}^{m\times d_{1}}$, patch features, and reconstruction features ${\text{F}}_{p},{\text{F}}_{r}\in {\mathbb{R}}^{m\times d_{3}}$ using SAC (14), CONCH (32), and the AD module, respectively. For text features, Quilt-LLaVA (15) is applied as a caption generation function *Gen*_*caption*_(**X**_*i*_) to produce patch descriptions, which are then encoded by the CONCH text encoder to obtain ${\text{F}}_{t}\in {\mathbb{R}}^{m\times d_{2}}$. The feature extraction methods are detailed in Section 3.3.

**Integrating multimodal features** Let **F** = (**F**_*p*_, **F**_*c*_, **F**_*t*_, **F**_*r*_) denote the quadruplet of feature matrices obtained from each modality, as described in Section 3.3. Given a MIL setup, our objective is to predict a bag-level label. Formally:


(13) $$\begin{array}{l} {\hat{\text{Y}}}_{i}=g\left(l\left(Select\left(\text{F}\right)\right)\right), \end{array}$$

where *g*(·) is a bag-level classifier and *Select*(·) is a selection function that returns a list of potential patches to be processed by the aggregation function *l*(·).

To integrate information from the different feature modalities, we design *l*(·) as a cascade of three cross-attention blocks (38). Each cross-attention operation is defined as:


(14) $$\begin{array}{l} \text{Attention}\left(\text{Q},\text{K},\text{V}\right)=\text{softmax}\left(\frac{\text{Q}{\text{K}}^{T}}{\sqrt{d_{k}}}\right)\text{V}, \end{array}$$

where *d*_*k*_ is the dimension of the key vectors.

For the first block, we incorporate textual information into cell features, which results **H**_1_ ← Attention(**F**_*t*_, **F**_*c*_, **F**_*c*_). In the second block, we integrate patch features with the result of the first block: **H**_2_ ← Attention(**H**_1_, **F**_*p*_, **F**_*p*_). In the third block, reconstructed patch features guide the integration, using **H**_2_ as keys and values: **H**_3_ ← Attention(**F**_*r*_, **H**_2_, **H**_2_). We then aggregate the instances by averaging the outputs of the third block: ${\bar{\text{H}}}_{3}=\frac{1}{m}\overset{m}{\underset{j=1}{\sum }}{\text{H}}_{3,j}$. Finally, we apply a linear layer to the aggregated *cls* token, ${\bar{\text{H}}}_{3}$, to obtain the $\text{logits}\leftarrow Linear\left({\bar{\text{H}}}_{3}\right)$ for the downstream tasks.

For classification, we use the Weighted Cross-Entropy loss to handle class imbalances:


(15) $$\begin{array}{l} L=-\frac{1}{b}\overset{b}{\underset{i=1}{\sum }}\overset{C}{\underset{c=1}{\sum }}w_{c},{\text{Y}}_{i,c}\log\left({\hat{\text{Y}}}_{i,c}\right), \end{array}$$

where $w_{c}=\frac{N_{\text{total}}}{C\cdot N_{c}}$, $N_{\text{total}}=\overset{C}{\underset{c=1}{\sum }}N_{c}$. Here, *w*_*c*_ is the weight for class *c*, *N*_*c*_ is the number of samples in class *c*, *C* is the total number of classes, and **Y**_*i,c*_ is the ground-truth label. This weighting scheme ensures that classes with fewer samples have a larger impact on the training process.

**Scaling AAMM with Perceiver IO** The standard Transformer architecture suffers from quadratic complexity *O*(*m*^2^) when attending over *m* instances, resulting in substantial computational and memory overhead. To alleviate this issue, we adopt the Perceiver IO framework (39), which replaces direct attention over the input space with attention over a latent space of fixed dimension.

**Scaling AAMM with reconstruction error** Beyond architectural optimization, we further reduce the computational load by selecting only a subset of instances for the feature extraction and full multimodal processing stages.

Given a fraction *k* ∈ (0, 1], the selected subset *S* has size ⌈*km*⌉. Consequently, the number of processed instances decreases by (1 − *k*) × 100%, significantly reducing computational costs while preserving critical information. Let **F**_*p*_ = {**f**_*p*,1_, …, **f**_*p,m*_} and **F**_*r*_ = {**f**_*r*,1_, …, **f**_*r,m*_} be the original and reconstructed features of the *m* instances from a given bag **X**, respectively. We define the reconstruction error for the *i*-th instance as $r_{i}=\|{\text{f}}_{p,i}-{\text{f}}_{r,i}{\|}_{2}^{2}$. Based on the set of reconstruction errors ${\left\{r_{i}\right\}}_{i=1}^{m}$, we consider two selection strategies.

1. *Maximum Selection:* Select the top-⌈*k*·*m*⌉ instances with the largest errors:


$$\begin{array}{r} \text{Given }\left\{r_{1},r_{2},\ldots ,r_{m}\right\},\text{let }\pi \text{ be a permutation of }\left\{1,2,\ldots ,m\right\}\\ \text{such that }r_{\pi \left(1\right)}\geq r_{\pi \left(2\right)}\geq \cdots \geq r_{\pi \left(m\right)}. \end{array}$$

Then, we define:


(16) $$\begin{array}{l} S_{\max}=\left\{\pi \left(1\right),\pi \left(2\right),\ldots ,\pi \left(\left\lceil km\right\rceil \right)\right\}. \end{array}$$

This strategy focuses on instances that are likely to contain tumor regions or hard-to-reconstruct patterns, thus potentially providing informative signals for classification.

2. *MinMax Selection:* Using the same sorted reconstruction scores *r*_π(1)_ ≥ *r*_π(2)_ ≥ ⋯ ≥ *r*_π(*m*)_, define $h=\frac{\left\lceil km\right\rceil }{2}$. The MinMax selection strategy then chooses the top *h* indices corresponding to the largest errors and the top *h* indices corresponding to the smallest errors:


(17) $$\begin{array}{r} S_{\text{minmax}}=\left\{\pi \left(1\right),\pi \left(2\right),\ldots ,\pi \left(h\right)\right\}\cup \\ \left\{\pi \left(m-h+1\right),\pi \left(m-h+2\right),\ldots ,\pi \left(m\right)\right\}. \end{array}$$

By including instances with both minimal and maximal reconstruction errors, this approach balances the feature space, prevents overconfidence, and can potentially reduce false negatives.

## 4 Experiments

### 4.1 Datasets

To evaluate the effectiveness of the proposed method, we conduct experiments on four histopathological datasets: CAMELYON16 (40), TCGA-LUAD (41), TCGA-LUSC (42), and SLN-Breast (43). The tasks include cancer classification and subtype classification. The TCGA-LUAD and TCGA-LUSC datasets are combined into a unified dataset for cancer subtype classification, which is referred to as TCGA-Lung. The datasets are preprocessed by dividing each WSI into 1024×1024 non-overlapping patches. Patches are extracted at magnifications of 40× for CAMELYON16, 20× for TCGA-Lung, and 40× for SLN-Breast. Before further processing, Macenko color normalization (44) is applied to address staining variability, and patches with more than 30% background are removed to enhance data quality. The CAMELYON16 dataset contains 398 WSIs, divided into 569,533 patches, while TCGA-Lung comprises 1,042 WSIs, resulting in 729,193 patches. Similarly, the SLN-Breast dataset contains 130 WSIs, divided into 29,497 patches. All three datasets are split into 5-fold cross-validation, with a standalone test set allocated for final performance evaluation. For CAMELYON16, the official test set is used. For TCGA-Lung, we adopt the test set provided in the DSMIL GitHub repository (21) to ensure fair comparisons. For SLN-Breast, we randomly selected 20% of the slides from the entire dataset to construct a testing set, maintaining the same distribution of negative and positive instances as the original dataset. The training and testing splits are in a ratio of 269:129 for CAMELYON16, 828:214 for TCGA-Lung, and 104:26 for SLN-Breast.

### 4.2 Baseline methods

We compare our method with nine baselines. Specifically,

- **Mean pooling** aggregates instance embeddings ${\left\{{\text{h}}_{i}\right\}}_{i=1}^{m}$ by computing the average $\text{z}=\frac{1}{m}\overset{m}{\underset{i=1}{\sum }}{\text{h}}_{i}$, where **h**_*i*_ can be the patch features or the concatenated feature vector of patch-cell-text features, depending on the uni- or multimodal settings.
- **Max pooling** selects the most salient instance $\text{z}\leftarrow \max_{i=1,\ldots ,m}{\text{h}}_{m}$ by taking the element-wise maximum, generating a fixed-size bag-level representation for classification.
- **ABMIL** (19) proposes the attention-based pooling mechanism for MIL, where a neural network assigns trainable weights to instances in a bag, enabling the aggregation of instance embeddings into a bag-level representation.
- **CLAM-SB/CLAM-MB** (20) is a weakly-supervised deep learning method that uses attention-based learning to assign weights to sub-regions of a whole slide for accurate classification and applies instance-level clustering to refine the feature space based on the most diagnostically relevant regions.
- **DSMIL** (21) combines a dual-stream MIL framework with self-supervised contrastive learning and multiscale feature fusion, where one stream uses max pooling to identify a critical instance and the other stream measures instance-to-critical-instance similarity to aggregate instance embeddings into a bag-level embedding.
- **TransMIL** (22) introduces a transformer-based MIL framework, leveraging self-attention to model correlations between instances and employing a Transformer Pyramid Position Encoding Generator (PPEG) to integrate spatial and morphological information.
- **ILRA-MIL** (45) uses low-rank matrix to capture global features and identify relationships between instances and incorporates a pathology-specific contrastive loss (LRC) to improve feature representation and classification performance.
- **MFMF** (16) uses the vanilla VAE as the backbone of the AD module and adopts the conventional cross-entropy loss for optimization.

To demonstrate the robustness of our method, we evaluate it against these baselines in both unimodal and multimodal settings. To ensure fairness, all methods use the same input features.

### 4.3 Evaluation metrics

We evaluate the performance of our proposed AAMM approach and nine baseline methods on the WSI classification task using 5-fold cross-validation. To ensure a comprehensive evaluation, we report the mean and standard deviation for three metrics: Area Under the Curve (AUC), Accuracy, and Recall. These metrics provide a comprehensive view of the model's ability to distinguish between classes, maintain overall correctness, and correctly identify positive instances. The AUC metric evaluates the discriminative capacity of the model across various classification thresholds, which is defined as:


(18) $$\begin{array}{l} \text{AUC}=\int_{0}^{1}TPR\left(FPR^{-1}\left(x\right)\right)dx. \end{array}$$

$TPR=\frac{TP}{TP+FN}$ and $FPR=\frac{FP}{FP+TN}$ are the True Positive Rate and False Positive Rate, respectively, and *TP, TN, FP, FN* denote the counts of True Positives, True Negatives, False Positives, and False Negatives.

Accuracy is another key metric, which quantifies the overall correctness of the classifier by computing the proportion of samples classified correctly among all samples.


(19) $$\begin{array}{l} \text{Accuracy}=\frac{TP+TN}{TP+TN+FP+FN}, \end{array}$$

Lastly, Recall measures the model's ability to identify positive cases, which is defined as:


(20) $$\begin{array}{l} \text{Recall}=\frac{TP}{TP+FN}, \end{array}$$

A higher Recall indicates better detection of positive samples.

## 5 Results

We conduct experiments using both unimodal and multimodal settings for a comprehensive analysis of the model's performance under varying input modalities. In the unimodal configuration, the input to all baselines is a set of patch feature vectors **F**_*p*_, which are extracted using the foundation CONCH model. For the proposed AAMM model, these **F**_*p*_ serve as the key and value inputs, while the reconstruction features **F**_*r*_ are used as the query. The two features are integrated through a single Perceiver IO block.

In the multimodal setting, the input comprises tuples of image, cell, and text features, denoted as **F**_*p*_ + **F**_*p*_ + **F**_*p*_ in the results tables. To adapt the multimodal setup for the baseline methods, these features are concatenated following the approach described in (23). For training the Abnormal Detection (AD) module, the normal class from CAMELYON16, the LUSC class from TCGA-Lung, and the negative class from SLN-Breast are designated as the training data.

### 5.1 Quantitative results

We first evaluate the performance of WSI classification on the CAMELYON16 dataset, where the results are shown in Table 1. In the unimodal setting, our AAMM variants consistently outperform or match the strongest baselines. Specifically, AAMM (MinMax) achieved an AUC of 0.9436, an accuracy of 0.9364, and a recall of 0.9179, surpassing most competing methods and highlighting its strong capability to correctly identify positive instances. AAMM (Max) and AAMM w/o Top-*k* also demonstrated robust performance, with AUC values above 0.94 and high recall scores. Compared with our conference version, AAMM with GMVAE achieves an AUC of 0.9432, higher than the 0.9402 obtained MFMF with VAE.

**Table 1:** Classification performance comparison on CAMELYON16.

**Feature**	**F**_*****p*****_			**F**_*****p*****_ **+**F****_*****c*****_ **+**F****_*****t*****_		
**Method**	**AUC**	**Accuracy**	**Recall**	**AUC**	**Accuracy**	**Recall**
Mean Pooling	0.6371 _±0.027_	0.7023 _±0.009_	0.6200 _±0.011_	0.6194 _±0.028_	0.5953 _±0.026_	0.5346 _±0.031_
Max Pooling	0.8018 _±0.012_	0.7721 _±0.006_	0.7087 _±0.008_	0.6831 _±0.044_	0.5581 _±0.137_	0.5607 _±0.074_
ABMIL	0.9302 _±0.003_	0.9108 _±0.008_	0.7837 _±0.021_	0.7711 _±0.011_	0.8031 _±0.013_	0.5020 _±0.010_
CLAM-SB	0.9233 _±0.001_	0.9077 _±0.008_	0.7633 _±0.016_	0.7446 _±0.024_	0.7769 _±0.034_	0.4449 _±0.047_
CLAM-MB	0.9092 _±0.012_	0.8954 _±0.010_	0.7265 _±0.021_	0.7417 _±0.018_	0.7538 _±0.019_	0.4000 _±0.031_
DSMIL	0.9334 _±0.003_	0.9339 _±0.006_	0.8367 _±1.1*e*−16_	0.8090 _±0.027_	0.8154 _±0.026_	0.6000 _±0.042_
TransMIL	0.9373 _±0.003_	0.9132 _±0.013_	0.8928 _±0.012_	0.8283 _±0.027_	0.8248 _±0.023_	0.7923 _±0.014_
ILRA-MIL	0.9402 _±0.006_	0.9400 _±0.003_	0.8489 _±0.010_	0.7742 _±0.050_	0.7723 _±0.059_	0.5469 _±0.076_
MFMF	0.9402 _±0.013_	0.9302 _±0.011_	0.9090 _±0.015_	0.9746 _±0.010_	0.9566 _±0.008_	0.9429 _±0.010_
AAMM w/o Top-*k*	0.9418 _±0.022_	0.9302 _±0.011_	0.9089 _±0.015_	*0.9576 _±0.005_*	*0.9488 _±0.011_*	0.9350 _±0.013_
AAMM (MinMax)	0.9436 _±0.022_	0.9364 _±0.013_	0.9179 _±0.019_	0.9768 _±0.006_	0.9504 _±0.013_	*0.9347 _±0.017_*
AAMM (Max)	0.9432 _±0.023_	0.9287 _±0.009_	0.9069 _±0.013_	**0.9773**_**±**0.007****_	**0.9597**_**±**0.006****_	**0.9469**_**±**0.008****_

The best result is shown in **bold**, the second-best result is underlined, and the third-best result is in *italics*. “AAMM *” represents our methods.

When integrating the multimodal features, the performance of AAMM is further improved. AAMM (Max) yields an AUC of 0.9773, an accuracy of 0.9597, and a recall of 0.9469, outperforming all other methods and demonstrating the effectiveness of incorporating multiple feature modalities. Notably, AAMM (MinMax) and AAMM w/o Top-*k* also attain superior results, indicating that the integration of cell-level and text-level information enhances the overall discriminative power of AAMM. AAMM with GMVAE improves performance across all metrics, achieving an AUC of 0.9773, accuracy of 0.9597, and recall of 0.9469 compared to MFMF's 0.9746, 0.9566, and 0.9429 respectively.

Table 2 shows the WSI classification results on the TCGA-Lung dataset. In the unimodal configuration, AAMM again demonstrates high performance. For instance, AAMM (Max) achieves an AUC of 0.9738, an accuracy of 0.9178, and a recall of 0.9183, surpassing most baseline methods and showing its robustness in a different pathological context. AAMM (MinMax) and AAMM w/o Top-*k* also maintain strong performance, with all variants consistently achieving high recall values. In the multimodal setting, AAMM (Max) and AAMM (MinMax) both reach an AUC of 0.9817. Among these, AAMM (Max) achieves the highest accuracy (0.9383) and recall (0.9385).

**Table 2:** Classification performance comparison on TCGA-Lung.

**Feature**	**F**_*****p*****_			**F**_*****p*****_ **+**F****_*****c*****_ **+**F****_*****t*****_		
**Method**	**AUC**	**Accuracy**	**Recall**	**AUC**	**Accuracy**	**Recall**
Mean pooling	0.9695 _±0.003_	0.9075 _±0.667_	0.9075 _±0.007_	0.9120 _±0.005_	0.8458 _±0.010_	0.8452 _±0.010_
Max pooling	0.9711 _±0.002_	0.9071 _±0.005_	0.9069 _±0.005_	0.8586 _±0.005_	0.7116 _±0.010_	0.7109 _±0.010_
ABMIL	0.9756 _±0.003_	0.9131 _±0.005_	0.8972 _±0.014_	0.9656 _±0.006_	0.9112 _±0.009_	0.9101 _±0.016_
CLAM-SB	0.9729 _±0.004_	0.9084 _±0.010_	0.9083 _±0.008_	0.9662 _±0.004_	0.9140 _±0.011_	0.9046 _±0.015_
CLAM-MB	0.9738 _±0.005_	0.9234 _±0.008_	0.9083 _±0.016_	0.9698 _±0.006_	0.9168 _±0.004_	0.9064 _±0.016_
DSMIL	0.9685 _±0.006_	0.9112 _±0.005_	0.9266 _±0.015_	0.9506 _±0.006_	0.8757 _±0.012_	0.9028 _±0.018_
TransMIL	0.9706 _±0.005_	0.9121 _±0.018_	0.9126 _±0.018_	0.9405 _±0.016_	0.8738 _±0.015_	0.8739 _±0.014_
ILRA-MIL	0.9742 _±0.006_	0.9206 _±0.008_	0.9276 _±0.025_	0.9531 _±0.005_	0.8869 _±0.012_	0.8844 _±0.018_
MFMF	0.9737 _±0.003_	0.9271 _±0.003_	0.9269 _±0.003_	0.9815 _±0.003_	0.9355 _±0.006_	0.9358 _±0.006_
AAMM w/o Top-*k*	0.9706 _±0.001_	0.9150 _±0.007_	0.9151 _±0.007_	0.9794 _±0.004_	*0.9215 _±0.010_*	*0.9217 _±0.011_*
AAMM (MinMax)	0.9744 _±0.003_	0.9140 _±0.008_	0.9142 _±0.008_	**0.9817**_**±**0.003****_	0.9355 _±0.006_	0.9358 _±0.006_
AAMM (Max)	0.9738 _±0.008_	0.9178 _±0.008_	0.9183 _±0.008_	**0.9817**_**±**0.003****_	**0.9383**_**±**0.005****_	**0.9385**_**±**0.005****_

The best result is shown in **bold**, the second-best result is underlined, and the third-best result is in *italics*. “AAMM *” represents our methods.

Table 3 shows the WSI classification results on the SLN-Breast dataset. In the unimodal configuration, AAMM exhibits strong performance, with AAMM (MinMax) achieving the highest AUC of 0.9865, outperforming the baseline methods and other AAMM variants. Similarly, AAMM (Max) and AAMM w/o Top-*k* also demonstrate competitive results, maintaining high accuracy and recall values, highlighting their robustness even without multimodal inputs.

**Table 3:** Classification performance comparison on SLN-Breast.

**Feature**	**F**_*****p*****_			**F**_*****p*****_ **+**F****_*****c*****_ **+**F****_*****t*****_		
**Method**	**AUC**	**Accuracy**	**Recall**	**AUC**	**Accuracy**	**Recall**
Mean Pooling	0.8850 _±0.000_	0.8846 _±0.000_	0.7857 _±0.000_	0.8308 _±0.041_	0.6385 _±0.301_	0.6714 _±0.139_
Max Pooling	0.8895 _±0.006_	0.8846 _±0.000_	0.7857 _±0.000_	0.8323 _±0.021_	0.7769 _±0.038_	0.7895 _±0.039_
ABMIL	0.9674 _±0.000_	0.9615 _±0.000_	0.8571 _±0.000_	0.9414 _±0.019_	0.8923 _±0.051_	0.8285 _±0.057_
CLAM-SB	0.9424 _±0.000_	0.9615 _±0.010_	0.8571 _±0.000_	0.9474 _±0.026_	0.9384 _±0.031_	0.8571 _±0.000_
CLAM-MB	0.9499 _±0.000_	0.9615 _±0.000_	0.8571 _±0.000_	0.9504 _±0.014_	0.9000 _±0.039_	0.7429 _±0.057_
DSMIL	0.9670 _±0.004_	0.8462 _±0.000_	0.8286 _±0.000_	0.9504 _±0.006_	0.8769 _±0.057_	0.7571 _±0.000_
TransMIL	0.9684 _±0.014_	0.9615 _±0.000_	0.9286 _±0.000_	0.9053 _±0.042_	0.8999 _±0.052_	0.8233 _±0.081_
ILRA-MIL	0.9774 _±0.000_	0.9615 _±0.024_	0.8571 _±0.090_	0.9534 _±0.006_	0.9077 _±0.019_	0.8571 _±0.000_
MFMF	0.9749 _±0.001_	0.9615 _±0.000_	0.9286 _±0.002_	0.9849 _±0.001_	0.9615 _±0.005_	0.9286 _±0.004_
AAMM w/o Top-*k*	0.9749 _±0.000_	0.9615 _±0.024_	0.9286 _±0.045_	0.9939 _±0.007_	0.9615 _±0.018_	0.9286 _±0.035_
AAMM (MinMax)	0.9865 _±0.005_	0.9615 _±0.019_	0.9286 _±0.035_	**0.9970**_**±**0.006****_	**0.9646**_**±**0.000****_	**0.9314**_**±**0.000****_
AAMM (Max)	0.9839 _±0.023_	0.9615 _±0.024_	0.9286 _±0.045_	*0.9920 _±**0.015**_*	0.9615 _±0.000_	0.9286 _±0.000_

The best result is shown in **bold**, the second-best result is underlined, and the third-best result is in *italics*. "AAMM *" represents our methods.

In the multimodal setting, AAMM (MinMax) achieves the best performance, with an AUC of 0.9970, an accuracy of 0.9646, and a recall of 0.9314. These results surpass all competing methods, including the second-best MFMF, which achieves an AUC of 0.9849. AAMM (Max) and AAMM w/o Top-*k* also maintain strong performance, with AUCs of 0.9920 and 0.9939, respectively. The results confirm that the integration of multimodal features and the application of Top-*k* selection strategies, particularly MinMax, significantly enhance classification accuracy and robustness in capturing abnormalities within all three observed datasets.

Furthermore, Tables 1–3 demonstrate that most SOTA methods achieve comparable performance on both the CAMELYON16, TCGA-Lung, and SLN-Breast datasets in unimodal settings. However, despite their complex architectures, these models struggle to maintain their performance when processing multimodal features. Traditional integration approaches, such as feature concatenation, fail to effectively manage scenarios where only one feature type (e.g., image) performs well while others introduce noise. Similarly, on the TCGA-Lung dataset, in the unimodal setting, AAMM achieves an AUC of 0.9738, very slightly higher than MFMF's 0.9737. In the multimodal setting, AAMM outperforms MFMF across all metrics, with an AUC of 0.9817 vs. 0.9815, accuracy of 0.9383 vs. 0.9355, and recall of 0.9385 vs. 0.9358.

The proposed AAMM approach consistently achieves higher recall scores compared to other methods. This is a crucial advantage in medical applications, as higher recall reduces the likelihood of missing critical abnormalities. Additionally, the substantial improvements observed when utilizing multimodal features highlight the importance of integrating diverse data representations. By leveraging multiple information streams, AAMM enhances the model's overall classification performance and reliability, demonstrating its potential for broader applications in histopathological image analysis.

### 5.2 Qualitative results

**Figure 2:**
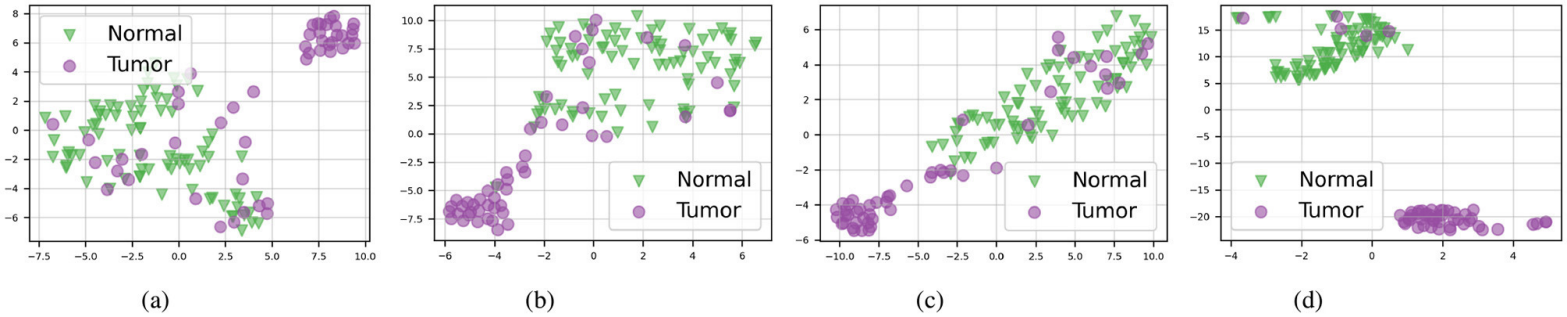
t-SNE visualizations of the CAMELYON16 test set in multimodal mode. **(A)** ABMIL. **(B)** TransMIL. **(C)** ILRA-MIL. **(D)** AAMM (ours).

To demonstrate the robustness of our approach, we visualize bag embeddings generated by four different methods on the CAMELYON16 test set under multimodal conditions. As shown in Figure 2, the AAMM model's scatter plot distinctly separates the ‘Normal' and ‘Tumor' categories, highlighting its superior capability in differentiating tissue types. In contrast, the other methods exhibit greater category overlap, indicating less effective feature integration and classification performance.

**Figure 3:**
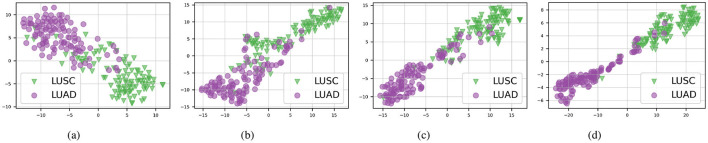
t-SNE visualizations of the TCGA-Lung test set in multimodal mode. **(A)** ABMIL. **(B)** TransMIL. **(C)** ILRA-MIL. **(D)** AAMM (ours).

Furthermore, as illustrated in Figure 3, our proposed method continues to outperform others on the TCGA-Lung test set in distinguishing between the two subtypes, “LUAD” and “LUSC”, with each category forming clearly defined clusters. This consistent performance across different datasets emphasizes the effectiveness of the AAMM model in feature integration and classification tasks, highlighting its potential for broader applications in histopathological image analysis.

### 5.3 Ablation study

#### 5.3.1 Observations

We analyze the robustness of each feature type (i.e., image, cell, text) in classification tasks, providing deeper insights into the structure of AAMM. The classification performance of TransMIL for different modalities on both datasets shows that patch features **F**_*p*_ substantially outperform other modalities. From Table 4, we observe that patch embeddings effectively capture the critical spatial and structural information present in histopathological images, leading to better classification outcomes. In contrast, cell and text embeddings show lower performance due to their inability to capture such detailed information.

**Table 4:** Classification performance of TransMIL for different modalities on CAMELYON16 (left) and TCGA-Lung (right).

**Feat**.	**AUC**	**Accuracy**	**Recall**	**AUC**	**Accuracy**	**Recall**
**F**_*p*_	0.9373 ± 0.003	0.9132 ± 0.013	0.8928 ± 0.012	0.9706 ± 0.004	0.9121 ± 0.017	0.9126 ± 0.018
**F**_*c*_	0.7245 ± 0.032	0.7643 ± 0.009	0.7135 ± 0.006	0.8656 ± 0.017	0.7832 ± 0.021	0.7823 ± 0.022
**F**_*t*_	0.6406 ± 0.008	0.6667 ± 0.031	0.6016 ± 0.019	0.7859 ± 0.023	0.7131 ± 0.033	0.7147 ± 0.033

In text descriptions, most prepositions and conjunctions may not be important for the tasks; only words related to tumors might be crucial. Additionally, the generative patch description sometimes contains a lot of unnecessary or noisy information. Here is an example of a generative patch description for a WSI with ID: tumor111 in CAMELYON16: “The image presents a clear view of the bone marrow. The most striking feature is the presence of cells that are identified as megakaryocytes. These are large, multinucleated cells that are responsible for the production of platelets, which are crucial for blood clotting. The presence of these cells, along with the absence of any abnormal or neoplastic cells, suggests that this is a healthy bone marrow sample. There is no evidence of a tumor or any other pathological condition in this patch.” Similarly, here is an example of a generative patch description for a WSI with ID: TCGA-6A-AB49-01Z-00-DX1 in TCGA-LUSC: “The image presents a complex scenario, with features that could be indicative of either lung adenocarcinoma or squamous cell carcinoma. The tissue architecture shows some ambiguity, making it challenging to definitively categorize the pathology.” Regarding cell-level features, intuitively, each WSI contains thousands of cells, but only a small portion of tumor-related cells contribute to the classification tasks.

#### 5.3.2 Top-*k* selection

**Improving performance:** We conducted a grid search experiment to further evaluate the Maximum and MinMax selection strategies, with the results summarized in Tables 5–7. On the CAMELYON16 dataset, the Maximum selection strategy slightly outperforms MinMax at certain *k* values. For instance, at *k* = 0.3, Maximum selection achieves an AUC of 0.9773 compared to MinMax's 0.9687, while at *k* = 0.2, MinMax selection nearly matches the performance of Maximum selection (AUC = 0.9768).

**Table 5:** Classification performance for different instance selection strategies on CAMELYON16.

	**Maximum selection**			**MinMax selection**		
**Top-** *k*	**AUC**	**Accuracy**	**Recall**	**AUC**	**Accuracy**	**Recall**
*k* = 0.4	0.9648 ± 0.0124	0.9442 ± 0.0158	0.9265 ± 0.0208	0.9708 ± 0.0109	0.9426 ± 0.0267	0.9245 ± 0.0351
*k* = 0.3	0.9773 ± 0.0072	0.9597 ± 0.0058	0.9469 ± 0.0076	0.9687 ± 0.0090	0.9426 ± 0.0222	0.9245 ± 0.0293
*k* = 0.2	0.9672 ± 0.0086	0.9488 ± 0.0144	0.9334 ± 0.0197	0.9768 ± 0.0062	0.9504 ± 0.0126	0.9347 ± 0.0166
*k* = 0.1	0.9606 ± 0.0193	0.9380 ± 0.0130	0.9215 ± 0.0154	0.9627 ± 0.0108	0.9442 ± 0.0237	0.9273 ± 0.0316

**Table 6:** Classification performance for different instance selection strategies on TCGA-Lung.

	**Maximum selection**			**MinMax selection**		
**Top-** *k*	**AUC**	**Accuracy**	**Recall**	**AUC**	**Accuracy**	**Recall**
*k* = 0.8	0.9769 ± 0.0038	0.9299 ± 0.0029	0.9303 ± 0.0028	0.9744 ± 0.0046	0.9168 ± 0.0075	0.9168 ± 0.0075
*k* = 0.6	0.9817 ± 0.0025	0.9383 ± 0.0054	0.9385 ± 0.0054	0.9817 ± 0.0027	0.9355 ± 0.0062	0.9358 ± 0.0058
*k* = 0.4	0.9769 ± 0.0038	0.9308 ± 0.0019	0.9312 ± 0.0018	0.9736 ± 0.0018	0.9206 ± 0.0066	0.9209 ± 0.0069
*k* = 0.2	0.9762 ± 0.0018	0.9280 ± 0.0023	0.9286 ± 0.0022	0.9639 ± 0.0097	0.9001 ± 0.0179	0.8999 ± 0.0183

**Table 7:** Classification performance for different instance selection strategies on SLN-Breast.

	**Maximum selection**			**MinMax selection**		
**Top-** *k*	**AUC**	**Accuracy**	**Recall**	**AUC**	**Accuracy**	**Recall**
*k* = 0.4	0.9774 ± 0.0415	0.9385 ± 0.0188	0.8857 ± 0.0350	0.9865 ± 0.0120	0.9615 ± 0.0000	0.9286 ± 0.0000
*k* = 0.3	0.9744 ± 0.0335	0.9538 ± 0.0154	0.9143 ± 0.0286	0.9835 ± 0.0262	0.9615 ± 0.0000	0.9286 ± 0.0000
*k* = 0.2	0.9920 ± 0.0155	0.9615 ± 0.0000	0.9286 ± 0.0000	0.9970 ± 0.0060	0.9646 ± 0.0000	0.9314 ± 0.0000
*k* = 0.1	0.9263 ± 0.0354	0.9154 ± 0.0923	0.8429 ± 0.1714	0.9549 ± 0.0463	0.9154 ± 0.0923	0.8429 ± 0.1714

For the TCGA-Lung dataset, both strategies achieve their best performance at *k* = 0.6, with an AUC of 0.9817. Maximum selection shows slightly higher accuracy (0.9383) and recall (0.9385) compared to MinMax (accuracy: 0.9355, recall: 0.9358). As *k* decreases, MinMax exhibits greater performance variability, with its AUC dropping to 0.9639 at *k* = 0.2, whereas Maximum remains more stable at 0.9762.

In the SLN-Breast dataset, MinMax selection consistently outperforms Maximum selection, particularly at *k* = 0.2, where MinMax achieves an AUC of 0.9970 compared to Maximum's 0.9920, while maintaining strong accuracy (0.9646) and recall (0.9314). For all three datasets, even at less optimal *k* values, both selection strategies remain competitive with baseline approaches. This overall robustness highlights the reliability and effectiveness of our instance selection strategy.

**Mitigating the noise problem in text features:** While text features add value, their use introduces potential challenges, primarily arising from errors in text generation and feature extraction. Text descriptions may contain noise or irrelevant information, as discussed in Section 5.3.1. For instance, unnecessary details about non-pathological elements can dilute the utility of these features. Additionally, if the text encoder fails to effectively filter out unimportant words, irrelevant information may propagate through the classification pipeline, further impacting performance. To address these issues, the proposed Top-*k* selection strategies mitigate noise by selecting potential patches based on their reconstruction errors, thereby reducing the likelihood of generating irrelevant or noisy text descriptions.

To evaluate the effectiveness of the Top-*k* selection strategies in reducing noise within text features, we conduct an experiment using only text features **F**_*t*_ with a single Perceiver IO block and compare the model's performance with and without the selection methods. Table 8 demonstrates that using the full text embeddings without selection methods results in lower performance compared to employing selection methods. This result indicates that by selecting the patches with the highest reconstruction errors, which are likely to contain clear abnormal information, Top-*k* selection helps reduce the number of irrelevant patches and enhances the overall performance of the system. The Max strategy consistently outperforms both the MinMax and W/o Top-*k* approaches across all datasets, particularly on SLN-Breast, where the AUC improves from 0.9135 to 0.9361. These findings validate the hypothesis that Top-*k* selection significantly enhances the utility of text features by prioritizing potential inputs.

**Table 8:** Performance comparison of WSI classification using text features **F**_*t*_ with and without Top-*k* selection strategies.

**Selection strategy**	**CAMELYON16**			**TCGA-lung**			**SLN-breast**		
	**AUC**	**Acc**.	**Recall**	**AUC**	**Acc**.	**Recall**	**AUC**	**Acc**.	**Recall**
W/o Top-*k*	0.6048	0.6744	0.6197	0.8108	0.7429	0.7499	0.9135	0.8654	0.7500
MinMax	0.6635	0.7364	0.6807	0.8110	0.7617	0.7617	0.9211	0.8846	0.7857
Max	0.6727	0.7442	0.6949	0.8056	0.7617	0.7622	0.9361	0.9038	0.8440

#### 5.3.3 Abnormal detection module

**Improving performance:** The primary motivations for incorporating the AD module are twofold: first, to reduce the number of processing instances, as demonstrated in the previous table; and second, to improve performance across key metrics. The results of our ablation experiments, detailed in Tables 9, 10 below, show that the inclusion of the AD module not only reduces computational costs but also significantly enhances the Recall metric, which is essential for reliable WSI classification in medical applications. To the best of our knowledge, this is the first work to apply reconstruction features **F**_*r*_ as queries (**Q**) in a cross-attention mechanism, enhancing WSI-based cancer classification by using reconstruction errors to specifically target tumor instances, which are scarce and limited in the dataset.

**Table 9:** Performance of AAMM w/o Top-*k* with different feature combinations on CAMELYON16.

**Feature**	**AUC**	**Accuracy**	**Recall**	**Precision**
**F**_*p*_ + **F**_*c*_	0.9326 ± 0.0202	0.9164 ± 0.0114	0.8987 ± 0.0165	0.9097± 0.0061
**F**_*p*_ + **F**_*c*_ + **F**_*r*_	0.9513 ± 0.0145	0.9411 ± 0.0167	0.9272 ± 0.0182	0.9294 ± 0.0189
**F**_*p*_ + **F**_*t*_	0.9420 ± 0.0081	0.9341 ± 0.0133	0.9152 ± 0.0179	0.9185 ± 0.0091
**F**_*p*_ + **F**_*t*_ + **F**_*r*_	0.9524 ± 0.0024	0.9380 ± 0.0126	0.9184 ± 0.0166	0.9431 ± 0.0087
**F**_*p*_ + **F**_*c*_ + **F**_*t*_	0.9478 ± 0.0159	0.9395 ± 0.0076	0.9236 ± 0.0113	0.9306 ± 0.0058
**F**_*p*_ + **F**_*c*_ + **F**_*t*_ + **F**_*r*_	0.9576 ± 0.0057	0.9488 ± 0.0105	0.9350 ± 0.0133	0.9582 ± 0.0090

**Table 10:** Performance of AAMM w/o Top-*k* with different feature combinations on TCGA-lung.

**Feature**	**AUC**	**Accuracy**	**Recall**	**Precision**
**F**_*p*_ + **F**_*c*_	0.9753 ± 0.0030	0.9190 ± 0.0075	0.9191 ± 0.0076	0.9149 ± 0.0077
**F**_*p*_ + **F**_*c*_ + **F**_*r*_	0.9777 ± 0.0024	0.9213 ± 0.0080	0.9213 ± 0.0081	0.9152 ± 0.0080
**F**_*p*_ + **F**_*t*_	0.9756 ± 0.0019	0.9211 ± 0.0091	0.9212 ± 0.0093	0.9120 ± 0.0089
**F**_*p*_ + **F**_*t*_ + **F**_*r*_	0.9785 ± 0.0033	0.9213 ± 0.0069	0.9215 ± 0.0065	0.9173 ± 0.0054
**F**_*p*_ + **F**_*c*_ + **F**_*t*_	0.9782 ± 0.0032	0.9211 ± 0.0087	0.9211 ± 0.0087	0.9215 ± 0.0085
**F**_*p*_ + **F**_*c*_ + **F**_*t*_ + **F**_*r*_	0.9794 ± 0.0041	0.9215 ± 0.0104	0.9217 ± 0.0106	0.9231 ± 0.0107

As shown in Table 9, adding the AD component to the AAMM model on CAMELYON16 increases the AUC from 0.9478 to 0.9576, accuracy from 0.9395 to 0.9488, and recall from 0.9236 to 0.9350. For TCGA-Lung (Table 10), the AUC rises from 0.9782 to 0.9794, accuracy from 0.9211 to 0.9215, and recall from 0.9211 to 0.9217. These consistent numeric gains highlight the importance of AD component in improving the classification performance.

**Reducing computational cost:** One of our motivations is that obtaining instance-level annotations is time-consuming. To address this, our proposed AAMM reduces the number of processing instances required during text generation, text feature extraction, and cell feature extraction by employing Maximum/MinMax selection strategies. This reduction directly impacts computational cost. We compare and report data preprocessing times with and without the Abnormal Detection (AD) module in Table 11. Given that the total number of instances in CAMELYON16 and TCGA-Lung are $\overset{b}{\underset{i}{\sum }}m_{i}=$ 569,533 and $\overset{b}{\underset{i}{\sum }}m_{i}=$ 729,193, respectively, where *m*_*i*_ is the number of instances in the *i*-th bag, *k* ∈ [0, 1) represents the percentage of instances in the bag to be processed, and *b* is the number of bags in dataset. Although training the AD module and extracting *F*_*r*_ features adds some overhead, the time saved by not processing $\left(1-k\right)\times \overset{b}{\underset{i}{\sum }}m_{i}$ instances during cell and text feature extraction is substantial. For example, in the cell feature extraction step for the CAMELYON16 dataset (*k* = 0.3, maximum selection), we save approximately $\frac{\left(1-0.3\right)\times 569,533\times 3.6992}{batch}=\frac{1,474,771.1616}{16}$ seconds, which is approximately 25 hours, by not processing 398,673 instances using the top-*k* approach.

**Table 11:** Comparison of computational time and processing instances with and without the AD module.

**Step**	**W/o AD module**	**With AD module**	**Processing time per step/instance (second)**
Train AD module	No	Yes	128.4508 ± 43.470 (CAM16)
			147.3088 ± 40.563 (TCGA)
Compute reconstruction error and *F*_*r*_	No	$\overset{b}{\underset{i}{\sum }}m_{i}$	0.1054 ± 0.0509
Extract *F*_*c*_	$\overset{b}{\underset{i}{\sum }}m_{i}$	$\overset{b}{\underset{i}{\sum }}k\times m_{i}$	3.6992 ± 0.0599
Generate patch description	$\overset{b}{\underset{i}{\sum }}m_{i}$	$\overset{b}{\underset{i}{\sum }}k\times m_{i}$	2.1242 ± 0.1224
Extract *F*_*t*_	$\overset{b}{\underset{i}{\sum }}m_{i}$	$\overset{b}{\underset{i}{\sum }}k\times m_{i}$	0.0643 ± 0.0012

Additionally, AAMM is an attention-based model, and one well-known drawback of attention mechanisms is the quadratic complexity *m*^2^. By reducing the number of instances, the computational cost for training attention-based models is also reduced proportionally (i.e., reducing by the factor of *k*). In summary, the AD module efficiently reduces computational costs in both the preprocessing and training stages.

### 5.4 Discussion

Our framework is designed for versatility and flexibility, enabling the integration and use of features from a diverse array of foundation models. It utilizes features from models such as CONCH, SAC, and Quilt-LLaVA. As more powerful and robust foundation models emerge, they can be smoothly incorporated into our existing framework, further enhancing its capability and scope. Furthermore, our framework is flexible due to its inheritance of Perceiver IO's capacity to handle diverse types and sizes of input data. This feature facilitates the efficient processing of varied data modalities. Moreover, the layered structure of our AAMM enhances its adaptability, supporting a plug-and-play methodology that allows for the straightforward addition or removal of modalities without necessitating extensive redesigns. This flexibility ensures that the framework can be rapidly adapted to meet the specific demands of different applications, making it a robust and evolving solution in line with continuous advancements in foundation models and data processing technologies. In the future, we plan to establish a benchmark to evaluate the effectiveness of various SOTA foundation model combinations within our framework. This approach will help us continuously improve and adapt our method to enhance its performance in WSI classification tasks.

## 6 Conclusions

In this study, we introduced the Abnormality-Aware Multimodal (AAMM) framework to address the challenges of WSI classification in histopathology. The AAMM framework effectively leverages multimodal data to enhance the performance in both normal-tumor and cancer subtype classification tasks. The incorporation of a Gaussian Mixture Variational Autoencoder (GMVAE) for abnormality detection further improves computational efficiency and model accuracy by selectively focusing on the most relevant instances. Our comprehensive experiments on the CAMELYON16 and TCGA-Lung datasets demonstrate the superiority of the AAMM framework over SOTA methods. The results highlight the importance of combining diverse modalities and utilizing abnormality-guided instance selection for robust WSI analysis. Additionally, the framework's flexibility allows for the seamless integration of various foundation models, making it a scalable solution for future advancements in computational pathology.

## Data Availability

Publicly available datasets were analyzed in this study. This data can be found here: https://camelyon16.grand-challenge.org/Data/; https://portal.gdc.cancer.gov/projects/tcga-luad; https://portal.gdc.cancer.gov/projects/TCGA-LUSC; https://www.cancerimagingarchive.net/collection/sln-breast/.
